# Upcycling Wool Textile Waste by Slow Pyrolysis to Recover Nitrogen-Rich Bio-Oil and Bio-Char and CO-Rich Gas Using Bespoke Auger Reactor

**DOI:** 10.3390/molecules31162816

**Published:** 2026-08-13

**Authors:** Roozbeh Kalateh, Danmei Sun, Aimaro Sanna

**Affiliations:** 1 Institute of Mechanical, Process and Energy Engineering, School of Engineering and Physical Sciences, Heriot-Watt University, Edinburgh EH12 7NT, UK; 2 School of Textiles and Design, Heriot-Watt University, Scottish Borders Campus, Galashiels TD1 3HF, UK; d.sun@hw.ac.uk

**Keywords:** wool waste, biochar, pyrolysis, bio-oil analysis, auger reactor

## Abstract

The valorisation of textile wool waste through sustainable conversion technologies such as pyrolysis has gained increasing attention as an effective strategy to reduce textile waste, recover valuable resources, and support the transition toward a circular economy. Herein, we investigated the pyrolysis of processed wool textile waste in CO_2_ and N_2_ atmospheres to recover valuable products and reduce the environmental impact. Key factors such as the temperature, carrier gas type, feed size, condensation set-up, and reactor configuration were evaluated for their influence on product distribution and quality. Pyrolysis at 900 °C in the presence of CO_2_ led to greater gas formation (79 wt%), enhanced the stability and BET surface area of the char (10–12 wt%), and increased byproducts including phenol and indole in the bio-oil (13 wt%) product. CO made up over 65% of the gas at 900 °C due to the prevalence of the reverse (endothermic) Boudouard reaction, with the remnant gas made of CO_2_ (21%) and small amounts of NH_3_ (2%), HCN (0.8%) and SO_2_ (0.3%). This CO-rich gas could have industrial applications such as Fischer–Tropsch after conditioning and N/S removal. Moreover, the higher carbon content (82.5% at 900 °C) increased the stability of char produced with CO_2_ (compared to N_2_), making it suitable for soil enhancement (~10% N at 900 °C) or pollutant removal and allowing it to be categorised and marketed as biochar. Despite low-temperature pyrolysis (350 °C) not being efficient in decomposing the whole wool waste, a staged pyrolysis with an initial low-temperature stage was shown to be effective in separately removing bromine-rich compounds. In summary, this study provides insights into the thermal decomposition behaviour of wool and the influence of the reaction conditions and reactor type on product distribution.

## 1. Introduction

The textile industry is responsible for significant environmental impacts, contributing approximately 8–10% of global CO_2_ emissions and generating more than 92 million tonnes of textile waste annually, much of which is landfilled or incinerated [1,2,3,4,5]. To mitigate these impacts, policies promoting sustainable textile management and waste valorisation are being implemented worldwide, including the UK Sustainable Clothing Action Plan [6].

Among textile materials, wool presents additional environmental challenges due to its high-water demand during production and the extensive use of dyes and finishing chemicals. At the same time, wool contains significant amounts of nitrogen and sulphur from its keratin structure, making its thermochemical conversion fundamentally different from that of cellulosic or synthetic textiles. Pyrolysis offers a promising route for recovering carbon while influencing the partitioning of nitrogen- and sulphur-containing species among gas, liquid, and solid products. Understanding the effects of process conditions on product yields and elemental distribution is therefore essential for developing efficient and environmentally responsible valorisation strategies for wool textile waste.

Wool is a natural fibre with a complex heterogenous structure consisting mainly of a protein called keratin, which has a larger content of N (~25 wt%) and H (~12 wt%) compared to, e.g., lignocellulosic materials [7,8]. Keratin also has high concentrations of sulphur, which is mainly due to the existence of cystine. The main reason for the lower solubility and higher stability of wool compared to other proteins is the formation of cross-links of disulphide bonds in cystine [9]. Disposal is currently the most used method for handling the textile waste around the world, while recycling and reusing are promising alternatives, which could provide low-cost clothing for African nations and a reduction in carbon dioxide emissions. Despite this, several issues, such as brand dilution, economical and policy-related concerns, are hindering these waste management paths [10]. 

Thermochemical conversion processes, particularly pyrolysis, have been proposed as a promising approach for the valorisation of textile waste [11]. Pyrolysis involves the thermal decomposition of biomass at temperatures of approximately 500 °C under oxygen-deficient conditions, producing three main product fractions: non-condensable gases, biochar, and bio-oil. Based on the residence time of the feedstock within the reactor, pyrolysis can generally be classified into three categories: fast, intermediate, and slow pyrolysis [12]. The distribution and composition of the gaseous, solid, and liquid products are influenced by several operating and feedstock-related parameters, including reactor configuration, process temperature, heating rate, feedstock composition, particle size, and reactor pressure. Recent studies have demonstrated the feasibility of using pyrolysis for the valorisation of textile waste, for example, in the production of hydrogen and in the co-pyrolysis with sludge, which increased the hydrocarbon yield and lowered emissions [13,14]. Similarly, the pyrolysis of wool waste provides an opportunity to reduce environmental impacts associated with wool waste disposal and to produce useful byproducts and energy that can be utilised for further industrial applications [15,16,17,18]. Wen et al. (2017) investigated the pyrolysis behaviour of three different textile feedstocks—cotton, wool, and polyester—using small samples (<1.2 mm) in a CO_2_ atmosphere [19]. Thermogravimetric analysis coupled with Fourier-transform infrared spectroscopy (TG–FTIR) was conducted over a temperature range of 300–500 °C [19]. The results indicated that cotton exhibited the highest reactivity, with pyrolysis commencing at approximately 300 °C, followed by polyester. In contrast, wool was the least reactive feedstock and showed the lowest sensitivity to changes in temperature. Liu et al., 2024 studied the microwave co-pyrolysis of textile dyeing sludge and furfural residue, where the addition of the latter neutralised the strong alkalinity and inhibited the formation of nitrogen compounds [18]. Ciuffi et al., (2025) studied the slow pyrolysis of high- and low-N-content textile wastes in an auger type reactor (3 kg/h max capacity) at temperatures up to 600 °C [20]. About 50% of the carbon contained in the textile waste was recovered as anthracite, and the char from the high-N-content (6.2 wt%) textile waste was found to have a low BET (12 m^2^/g), while benzoic acid was the main compound found in all the condensates and can be extracted [20].

The application of dyes on wools for use, for example, in the fashion industry, changes the properties of the wool. Therefore, consideration of the textile dyes in the pyrolysis is essential. One categorisation method for dyes is based on the chemical structure. Acidic and reactive dyes are the main classes applied to wool. Reactive dyes normally have a molecular weight of 500 to 900 g/mol and contain a sulphonic acid group. Acid dyes themselves contain low-molecular-weight (300–400 g/mol) or high-molecular-weight (600–900 g/mol) compounds [21]. Understanding the pyrolysis process and the properties of the products derived from wool waste is essential for optimising its potential as a sustainable resource. However, the literature shows a lack of research on wool processed waste pyrolysis and its potential conversion into valuable biochar and recovery of potential nitrogen-rich chemicals. Therefore, this paper explores the pyrolysis of textile wool waste, highlighting the characteristics of the products obtained and the factors influencing the process, such as the temperature (350 (only auger reactor), 700, 800 and 900 °C), carrier gas (N_2_, CO_2_), and reactor type (fixed-bed or auger reactor). 

## 2. Results

### 2.1. Product Distribution

The product distribution obtained from the experiments conducted in the fixed-bed reactor is presented in Table 1. In general, increasing the pyrolysis temperature resulted in a greater release of volatile compounds and, consequently, a reduction in char yield. This increase in volatile production was particularly pronounced between 700 and 800 °C, as the main decomposition of untreated wool occurs predominantly between approximately 200 and 600 °C. More specifically, moisture begins to be released from wool at temperatures below approximately 150 °C. As the temperature increases, intermolecular and chemical bonds, including hydrogen bonds, disulphide (S–S) bonds associated with the cross-linking of wool proteins, and salt bonds, begin to break down at around 320 °C. Since the dissociation of C–C and C–H bonds requires approximately 35% more energy than that of S–S bonds, the degradation of the main macromolecular structure of wool occurs at approximately 430 °C [22]. The amount of oil produced at 800 °C in the presence of CO_2_ was less than that in the presence of N_2_. This can be explained by the reactivity of CO_2_ that resulted in enhanced cracking and gas formation (+5.7 wt%). Higher gas yields were generated by increasing the temperature from 700 to 900 °C (+26.7 wt%) in the presence of CO_2_, due to secondary reactions and char gasification in the presence of CO_2_ [23].

#### 2.1.1. Char Analysis

The char obtained from the pyrolysis of wool in the FB reactor could be categorised as brittle and flaky (Figure S1a). The effect of temperature on the BET of the chars obtained in the presence of CO_2_ was examined. Char produced from the pyrolysis of large wool pieces at 800 °C was selected to investigate the effect of the carrier gas on the Brunauer–Emmett–Teller (BET) surface area of the resulting char. BET surface areas of 113 ± 24 m^2^/g and 192 ± 16 m^2^/g were obtained using CO_2_ and N_2_ as the carrier gases, respectively. The higher surface area obtained under N_2_ may be attributed to differences in the interaction of the carrier gas with the char surface and the extent of secondary reactions occurring during pyrolysis. Although the BET surface area of the resulting char was below the threshold typically associated with activated carbon, it was higher than that reported for several other biomass-derived chars, including those produced from coconut shells, palm oil residues, and orange peels under standard conditions [24].

An increase in the pyrolysis temperature was also associated with a corresponding increase in the BET surface area of the char (Table 2). For example, char produced at 700 °C using CO_2_ as the carrier gas exhibited a BET surface area of 59 m^2^/g, whereas the char produced at 900 °C reached 190 m^2^/g. This temperature-dependent increase in surface area is consistent with previously reported trends in the literature [25].

Elemental analysis (EA) was used to determine the concentrations of carbon (C), hydrogen (H), nitrogen (N), and oxygen (O) in the solid residues. These data provide an indication of the degree of carbonisation of the char and its potential suitability for applications such as soil amendment. The carbon content of the solid product exceeded 70 wt% for chars produced from processed wool waste at temperatures above 700 °C, indicating a high degree of carbonisation. The relatively high protein content of the processed wool waste (15.9 wt%) also explains the presence of nitrogen in the resulting char.

Table 2 presents the effect of the pyrolysis temperature on the elemental composition of the char. Increasing the pyrolysis temperature resulted in an increase in the carbon content, accompanied by reductions in the hydrogen and nitrogen contents. This trend is consistent with observations reported in the literature [23] and can be attributed to the progressive devolatilisation and carbonisation of the feedstock at higher temperatures.

From a biochar utilisation perspective, the relatively high nitrogen content of the resulting char (11–15 wt%) could provide an advantage for soil amendment applications compared with conventional lignocellulosic biochars, potentially providing a source of nitrogen. However, this nitrogen content may be less favourable for fuel applications because nitrogen-containing compounds can contribute to NO_x_ formation during combustion.

The H/C ratio is an important parameter for assessing the stability and degree of carbonisation of the resulting char. In general, a lower H/C ratio indicates a greater degree of carbonisation, increased aromaticity, and greater chemical stability of the solid product. An H/C ratio below 0.7 is commonly used as an indicative threshold for classifying biochar as sufficiently stable [26]. The H/C ratio can also provide an indication of the aromaticity of biochar, which is closely related to its potential adsorption properties. As the pyrolysis temperature increases, the progressive removal of hydrogen-containing volatile compounds generally results in a lower H/C ratio and a more condensed aromatic structure.

All char samples exhibited H/C ratios below 0.5, indicating a relatively high degree of carbonisation. The chars produced at 800 and 900 °C exhibited particularly low H/C ratios of approximately 0.1. These low values suggest a highly carbonised and aromatic structure, which may enhance their potential for adsorption applications. In particular, the chars produced at higher temperatures could have potential for the removal of organic pollutants such as naphthalene and phenanthrene, as highly carbonised materials with low H/C ratios are generally associated with enhanced adsorption capacity. Similarly, the O/C ratio can be used to assess the degree of carbonisation and stability of biochar and to distinguish it from other solid pyrolysis products. The O/C ratios of all the samples were well below the threshold value of 0.4. The low O/C ratios indicate a high degree of carbonisation and a relatively stable carbon structure. Based on their H/C and O/C ratios, the chars produced from wool pyrolysis satisfy the commonly applied criteria for biochar classification and therefore demonstrate the potential for utilisation as biochar materials.

#### 2.1.2. Gas Analysis

The main gases produced in the experiments at 700, 800 and 900 °C are shown in Table 3. CO_2_ was the main product at 700 °C, while CO became the main gas product when the temperature increased to 800 and 900 °C. More precisely, at 900 °C, CO made up over 65% of the gas, while the CO_2_ content was only 21%. The pronounced increase in CO concomitant with the depletion of CO_2_ at 900 °C suggests enhanced CO_2_–char gasification. In particular, the Boudouard reaction (C + CO_2_ → 2CO) is expected to contribute substantially at this temperature. This reaction can also promote the development of the char pore structure through partial gasification of the carbon matrix, consistent with the observed increase in the BET surface area. However, the concurrent increase in H_2_O and the decrease in the oxygen content of the solid indicate that additional reactions, including dehydration/deoxygenation of oxygen-containing structures and steam–carbon reactions, also contribute to the high-temperature gas evolution. Therefore, the increase in CO should be considered the combined result of CO_2_ gasification and secondary solid–gas/gas-phase reactions, rather than being attributed exclusively to the Boudouard reaction. In addition to CO and CO_2_, relatively small quantities of C_1_–C_4_ hydrocarbons were detected at all investigated temperatures. However, their abundance decreased with increasing temperature, which can be attributed to enhanced secondary cracking reactions at higher temperatures. Since wool waste is predominantly protein-based and contains appreciable amounts of nitrogen and sulphur, the formation of nitrogen- and sulphur-containing compounds in the gaseous products was expected during pyrolysis. Furthermore, the decrease in the nitrogen content observed in the char at higher temperatures suggests that a greater proportion of nitrogen was released into the volatile products. As shown in Table 3, ammonia (NH_3_) was the predominant nitrogen-containing compound among those identified. Hydrogen cyanide (HCN) and dimethylamine (C_2_H_7_N) were also detected in quantifiable concentrations. The concentrations of both HCN and NH_3_ increased with increasing pyrolysis temperature, indicating a positive correlation between temperature and the formation of these nitrogen-containing gaseous products. This trend is consistent with the elemental analysis of the char produced under the corresponding pyrolysis conditions, which showed a decrease in nitrogen content at higher temperatures.

This CO-rich gas can be useful in industrial applications such as Fischer–Tropsch or methanol production, but since it contains nitrogen and sulphur poisons (NH_3_, HCN, SO_2_) at levels that would rapidly deactivate common synthesis catalysts used in those processes, the gas would need a conditioning train tailored to the target process.

Acetamide was also detected in the gaseous products, although only in relatively small quantities. The identified nitrogen-containing compounds are combustible and could potentially contribute to the energy content of the product gas when combusted alongside other combustible syngas components. However, their combustion may result in the formation of nitrogen oxides (NO_x_), which are undesirable atmospheric pollutants. Therefore, appropriate gas treatment would be required prior to combustion or atmospheric release. Since several of these nitrogen-containing compounds are water-soluble, wet scrubbing using water or an alkaline solution such as NaOH could be employed to remove them from the product gas. This treatment would reduce the release of nitrogen-containing pollutants and improve the environmental performance of the pyrolysis process. Previous studies have also demonstrated that nitrogen present in syngas, particularly in the form of NH_3_, can be recovered through scrubbing and subsequently reused for value-added applications, such as microalgae cultivation or fertiliser production [27]. This approach could therefore provide an opportunity for nitrogen recovery while simultaneously reducing potential atmospheric emissions.

#### 2.1.3. Oil Analysis

GC-MS was used to identify and provide a relative quantification of the chemical components of the oils. Generally, the most abundant products found in the oil samples were phenolics and indoles. This could be related to the structure of the dyes used in dying the wool, which can contain benzene rings. Another group of compounds identified in the pyrolysis products comprised simple ketones, such as 4-hydroxy-4-methyl-2-pentanone. The formation of these compounds may be associated with the breakdown of hydrogen bonds and isopeptide cross-links within the wool protein structure during thermal degradation.

Given the proteinaceous nature of wool and the presence of amino acids such as histidine and proline, the formation of nitrogen-containing compounds in the pyrolysis oil was expected. These compounds were detected in several chemical classes, including pyrazines, amines, amides, nitriles, and aziridines. The presence and diversity of these nitrogen-containing compounds reflect the decomposition of the proteinaceous components of wool during pyrolysis and highlight the distinct chemical characteristics of wool-derived pyrolysis oil compared with oils produced from lignocellulosic feedstocks. Previous work by Kalateh et al. (2024) showed that catalytic pyrolysis of the same wool waste enhanced the production of aromatics, indoles, pyrroles, and amides [28].

Overall, the number of compounds detected increased with increasing pyrolysis temperature. Across all samples, irrespective of the operating temperature, the 20 most abundant compounds identified by GC–MS accounted for more than 55% of the total detected products. Therefore, to facilitate a meaningful comparison between the different samples, the analysis was limited to these 20 most abundant compounds.

##### Effect of Condensation System and Carrier Gas

**Figure 1:**
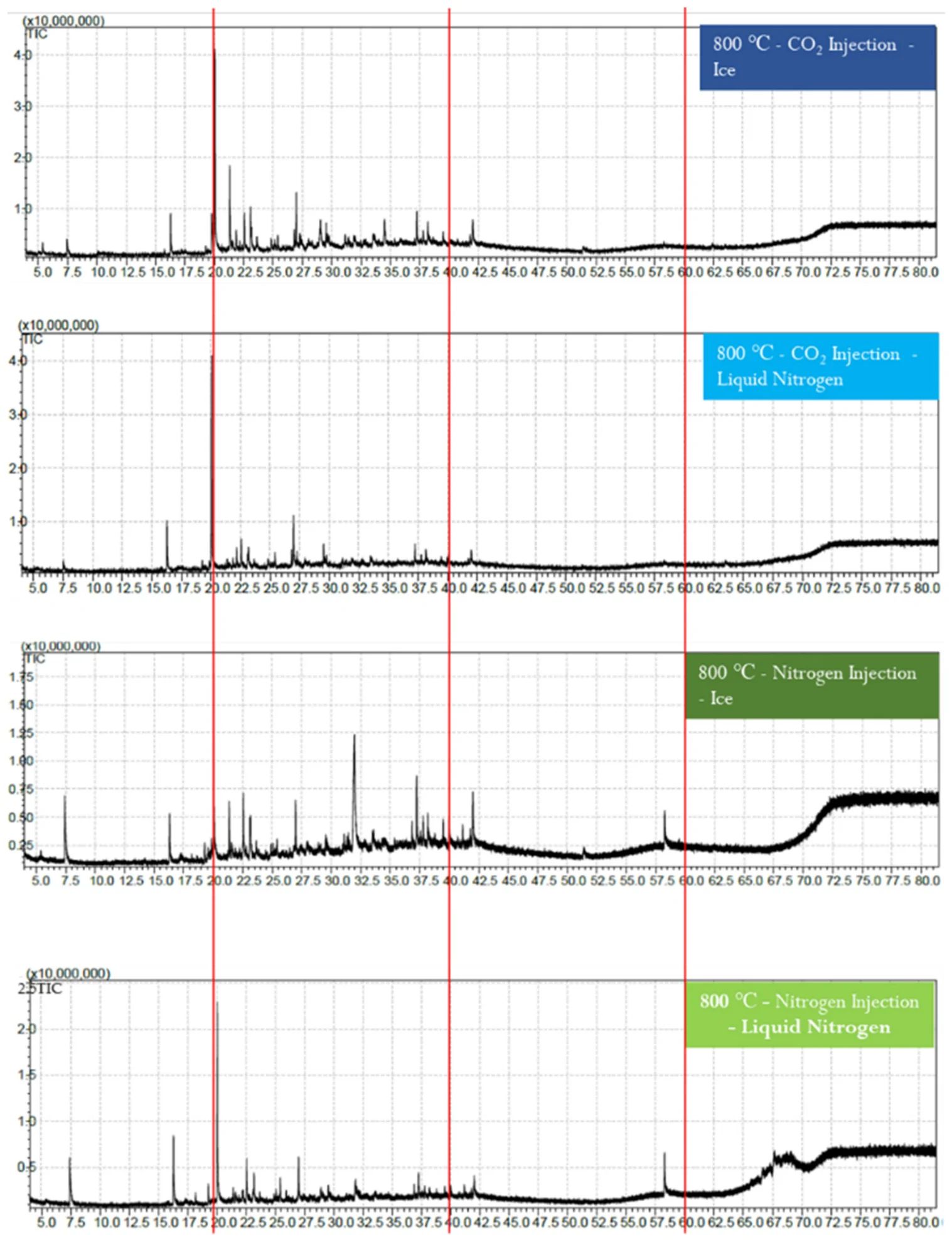
GC-MS chromatograms showing the effect of carrier gas and condensation system type on pyrolysis oil composition. Vertical red lines highlight C number groups 1–20, 20–40 and >40.

Figure 1 presents the GC–MS profiles obtained from pyrolysis at 800 °C using different carrier gases (N_2_ and CO_2_) and condensation media (liquid nitrogen and an ice–water mixture). When the ice–water mixture was used as the condensation medium, a shift in the distribution of the condensed compounds was observed depending on the carrier gas employed. However, this difference was not apparent when liquid nitrogen was used as the condensation medium. A further difference was observed in the number of unique compounds identified in the GC–MS analysis. For the N_2_ case, 166 unique compounds were detected, whereas more than 240 compounds were identified when CO_2_ was used as the carrier gas. This higher number of compounds suggests that CO_2_ participated in secondary reactions with the pyrolysis vapours, thereby altering the composition of the condensable products. The results are consistent with previous studies indicating that CO_2_ can promote the formation of monophenols while reducing the formation of methoxy-containing compounds [29,30]. This trend was also evident when comparing the overall relative abundance of phenolic, indolic, and methoxy-containing compounds. The use of CO_2_ as the carrier gas resulted in an approximately 2% increase in the relative abundance of phenolic compounds, accompanied by an approximately 1% decrease in methoxy-containing compounds. These changes further support the potential role of CO_2_ in modifying the composition of wool-derived pyrolysis vapours.

##### Effect of Temperature

The GC–MS profiles of the oil samples obtained from pyrolysis at 700, 800, and 900 °C (Figure S4) were analysed to investigate the effect of pyrolysis temperature on the composition of the oil products. Overall, the samples exhibited similar peak profiles, although differences in peak intensity were observed with increasing temperature. A substantial increase in the number of unique compounds was observed when the temperature increased from 700 °C, at which 141 compounds were identified, to 800 °C, at which more than 240 compounds were detected. However, a comparable increase was not observed when the temperature was further increased from 800 to 900 °C. This may be attributed to the increased promotion of gasification reactions at 900 °C, as indicated by the corresponding increase in gas yield presented in Table 1. The increase in the relative abundance of phenolic compounds was particularly pronounced between 700 and 800 °C, with an increase of approximately 10%, whereas only a further 2% increase was observed between 800 and 900 °C. In contrast, the increase in the abundance of indole compounds was more pronounced between 800 and 900 °C. To investigate the possible reasons for these temperature-dependent trends, the ten most abundant compounds identified in the bio-oils were compared, as presented in Table 4. This approach was considered appropriate because the 20 most abundant compounds accounted for more than 55% of the detected products, while the ten most abundant compounds represented more than 45% of the products. Increasing the pyrolysis temperature resulted in both an increase in the number of highly abundant compounds and changes in their relative composition. At all investigated temperatures, the compound with the largest GC–MS peak area belonged to the phenolic group. These compounds were predominantly cresol isomers, namely, o-cresol (2-methylphenol), m-cresol (3-methylphenol), and p-cresol (4-methylphenol). The increase in the abundance of methyl-substituted phenolic compounds with increasing temperature may be associated with the thermal decomposition of dye components present in the textile feedstock. For example, acid green dyes can contain aromatic structures with methyl substituents and sulphonate groups (–SO_3_Na). The gas analysis showed that the concentration of SO_2_ increased with increasing pyrolysis temperature. This may indicate the progressive decomposition of sulphur-containing components originating from the dyes and/or keratin, resulting in a greater transfer of sulphur to the gaseous phase at higher temperatures. The simultaneous increase in SO_2_ production and the abundance of cresol compounds may suggest that the thermal decomposition of sulphur-containing components influences the formation and distribution of methyl-substituted phenols. However, further investigation would be required to establish a direct relationship between sulphur removal and cresol formation. Indole was also among the most abundant compounds, particularly in the oil produced at 800 °C. The increasing abundance of nitrogen-containing compounds in the oil with increasing temperature is consistent with the elemental analysis of the char, which showed a corresponding decrease in the nitrogen content at higher temperatures. This suggests that a greater proportion of the nitrogen originally present in the wool was transferred from the solid phase to the volatile products as the pyrolysis temperature increased. The presence of indole and other nitrogen-containing compounds in the oil therefore provides further evidence of the progressive decomposition and redistribution of nitrogen-containing components within the wool-derived feedstock during pyrolysis.

### 2.2. Scale-Up Wool Waste Pyrolysis Tests Using a Bespoke Auger Reactor

To verify the feasibility of scaling up the wool waste pyrolysis in terms of product yield and composition, the pyrolysis was carried out using a customised auger reactor (AR). The reactor was designed to feed up to 1 kg/h wool waste, but the tests were carried out feeding 0.4 kg/h for 2 h each, due to operating and safety reasons. Pyrolysis was carried out at 350, 500, 800 and 900 °C with carbon dioxide and at 800 °C with nitrogen, to observe the effect of an increase in temperature and a change in the gas at a larger scale. 

#### 2.2.1. Process Considerations

The comparison between the auger reactor (AR) and semi-fixed-bed (FB) reactor was conducted with consideration of the fundamental differences between the two processes. Although both systems operated under nominally isothermal conditions, the scale-up was performed using a continuous configuration in the AR, whereas the FB experiments were conducted in a smaller-scale, batch arrangement. Consequently, several operational parameters differed between the two systems and may have influenced the distribution and composition of the pyrolysis products. One of the main differences was the residence time of the pyrolysis vapours within the heated zone. The estimated gas residence times in the small- and large-scale reactors were approximately 28 and 200 s, respectively. The residence time of the solid feedstock also differed substantially between the two systems. In the FB experiments, wool was introduced into the heated reactor once the desired operating temperature had been reached. The feedstock was then allowed to undergo pyrolysis until the process was considered complete, with the wool-derived char remaining at the target temperature for approximately 15 min before the reactor was cooled and dismantled. A similar procedure was initially adopted for the AR, with wool being introduced after the furnace had reached the desired operating temperature. However, owing to the continuous movement of the feedstock through the reactor, the nominal residence time of the wool was considerably shorter. The wool required approximately 35 s to travel through the heated section before being discharged as char/ash into the char receiver. Consequently, whereas the char produced in the FB remained exposed to the target temperature for approximately 15 min, the material in the AR experienced a substantially shorter residence time. The actual residence time in the AR may, however, have varied because of the formation of wool agglomerates, which could have been transported by the auger flights. In addition, some char accumulated between the auger flights, as illustrated in Figures S1b and S2, potentially increasing the residence time of portions of the solid material. Another important difference between the two systems was the efficiency and configuration of the condensation train. In the FB experiments, the relatively small quantity of feedstock (1 g) allowed the use of highly efficient condensation media, including ice–water mixtures and liquid nitrogen, to maximise the recovery of condensable products while requiring a relatively small heat-transfer area. In contrast, the use of liquid nitrogen or ice-based condensation systems at the larger scale was considered impractical and potentially costly for future commercial operation. Therefore, the AR was equipped with two Quickfit jacketed coil condensers, providing a larger heat-transfer surface area and allowing readily accessible tap water to be used as the cooling medium. The change in condensation conditions may have influenced the composition and recovery of the bio-oil obtained from the AR compared with the FB system. Differences in the cooling rate and condensation temperature can affect the recovery of compounds with different volatilities and may also promote secondary reactions within the vapour phase before condensation. Therefore, differences observed between the bio-oils produced by the two reactors should be interpreted in the context of the different condensation configurations. A further modification introduced in the AR system was the incorporation of a water-scrubbing stage for the product gas prior to its release into the atmosphere. This treatment was implemented to reduce the potential release of water-soluble and potentially harmful gaseous compounds, particularly nitrogen-containing species identified during the small-scale experiments. The composition of the scrubbed gas was subsequently analysed to assess the effectiveness of water scrubbing in removing these compounds and to determine the potential for recovering nitrogen-containing species from the gas stream. Overall, the differences in the reactor configuration, solid and vapour residence times, condensation conditions, and gas treatment should be considered when comparing the products obtained from the FB and AR experiments. These differences mean that the variations in product yield and composition cannot necessarily be attributed solely to reactor scale-up or reactor type but may also result from the associated changes in operating conditions and downstream product-recovery systems.

#### 2.2.2. Char Analysis

**Figure 2:**
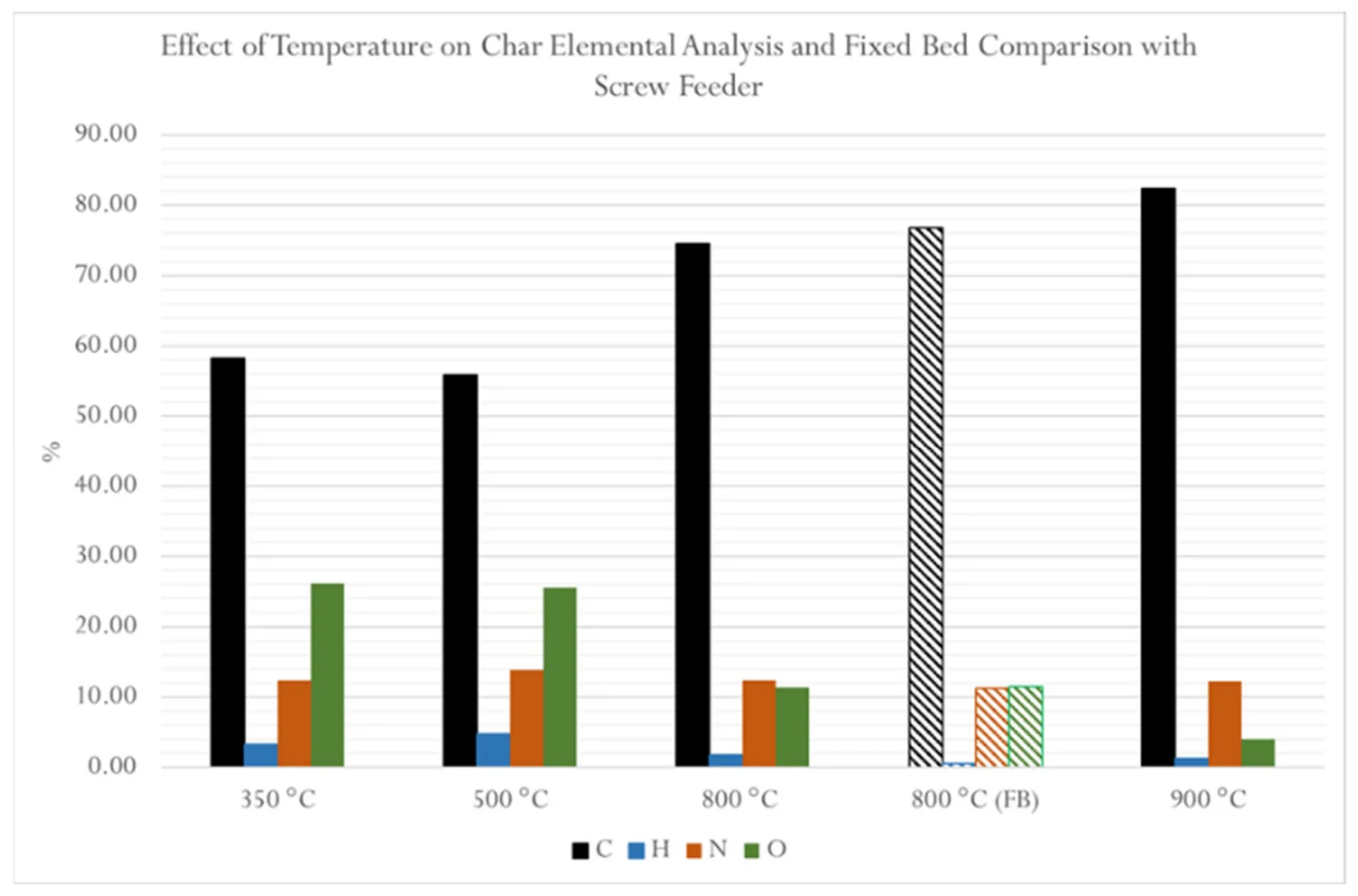
Elemental analysis of screw feeder char samples at different temperatures and fixed bed at 800 °C.

As observed for the fixed-bed experiments, the char produced in the auger reactor generally exhibited a brittle and flaky appearance. However, the sample obtained at 350 °C was an exception. At this temperature, the product appeared to be only partially pyrolysed, with the original wool structure still visible in the core of the char particles. The difference between the 350 °C samples obtained from the small- and large-scale reactors can be attributed to the longer residence time of the char in the small-scale fixed-bed reactor, which provided more time for the wool to undergo thermal decomposition. This behaviour was not observed at the higher pyrolysis temperatures, where the increased thermal severity resulted in more complete conversion of the wool. Figure 2 presents the elemental composition of the chars produced at different pyrolysis temperatures. The results obtained at 800 °C from both the auger and fixed-bed reactors are also presented to facilitate direct comparison between the two reactor configurations. As observed in the fixed-bed experiments, increasing the pyrolysis temperature resulted in an increase in the carbon content of the char, accompanied by decreases in the nitrogen and hydrogen contents. These trends are consistent with the progressive devolatilisation and carbonisation of the wool at higher temperatures. Two deviations from the general trends observed in the fixed-bed experiments were identified. The first relates to the oxygen content of the char. In the auger reactor, the oxygen content decreased with increasing pyrolysis temperature. This behaviour is consistent with the expected progression of thermal decomposition, whereby increasing the temperature promotes the release of oxygen-containing volatile compounds, resulting in a more highly carbonised and oxygen-depleted solid residue. A similar decrease in the oxygen content with increasing temperature was also observed for the chars produced in the fixed-bed reactor.

The sample obtained at 350 °C did not follow the trends observed for the samples produced at the higher temperatures, which can be attributed to the incomplete pyrolysis of the wool at this temperature, as discussed previously. A direct comparison between the chars produced in the fixed-bed and auger reactors was conducted using samples obtained at 800 °C. The char produced in the fixed-bed reactor contained approximately 2% more carbon and 1% less hydrogen than that produced in the auger reactor. These relatively small differences may be attributed to the longer residence time of the char at the final pyrolysis temperature in the fixed-bed reactor, which allowed further devolatilisation and carbonisation. Overall, the elemental analysis indicates that the two reactor configurations exhibited very similar behaviour, despite their differences in scale and operating mode.

#### 2.2.3. Scrubbing Water

To assess the potential removal of nitrogen- and sulphur-containing contaminants from the CO-rich gas for possible downstream applications, such as Fischer–Tropsch (F–T) synthesis, the effectiveness of cost-effective water scrubbing was investigated using gas produced in the auger reactor. Since the elemental composition of the auger reactor char was similar to that of the fixed-bed char, a comparable volatile composition was expected, with differences mainly attributed to the condensation system. GC–MS analysis of the scrubbing water was conducted to identify the compounds removed from the gas. As shown in Figure S2, small wool particles were entrained in the gas stream and either accumulated on the water surface or settled at the bottom of the scrubber, suggesting that a cyclone or similar particulate-separation device should be incorporated into an improved system. The colour of the scrubbing water changed from transparent to different shades of yellow depending on the pyrolysis temperature. Significant gas accumulation was also observed above the water, while pale-yellow condensate appeared in the outlet tubing. These observations suggest that the condensation system was not fully effective and that increasing the heat-transfer area and/or reducing the condensation temperature could improve bio-oil recovery. Figure S3 presents the GC–MS profile of the scrubbing water obtained at 800 °C. Fewer major compounds were detected compared with the corresponding bio-oil. Notably, 3,3′-thiobis propanenitrile (C_6_H_8_N_2_S), a toxic nitrogen- and sulphur-containing compound, accounted for more than 25% of the identified compounds in the scrubbing water, compared with less than 1% in some of the bio-oil samples. Other nitrogen-containing compounds, including pyrrolo[1,2-a]pyrazine-1,4-dione and hexahydro-3-(2-methylpropyl), were also detected. Their presence indicates that water scrubbing was effective to some extent in transferring potentially hazardous nitrogen- and sulphur-containing species from the gas phase into the aqueous phase. The yellow coloration of the scrubbing water may also be associated with the presence of 3,3′-thiobis propanenitrile, which is reported to be colourless to pale yellow. In addition, compounds commonly detected in the condensed bio-oil, including acetic acid, p-cresol, butanedinitrile, propanoic acid, and undecane, were also identified in the scrubbing water, although at considerably lower concentrations. Their presence indicates that some condensable compounds were passing through the condensation train and being captured by the scrubber. Therefore, improvements to the condensation system, potentially through increased heat-transfer area or lower operating temperatures, are required to maximise bio-oil recovery and minimise the transfer of organic compounds to the scrubbing water. The resulting contaminated scrubbing water may otherwise introduce additional treatment and disposal requirements.

#### 2.2.4. Bio-Oil Analysis

Table 5 presents the elemental composition of the bio-oils produced under different conditions using the auger reactor. The elemental compositions of the top and bottom oil fractions obtained at 350 °C showed no significant differences. Overall, the effect of increasing temperature was similar to that observed for the char. Increasing the pyrolysis temperature generally resulted in an increase in the carbon content and a decrease in the oxygen content, with the exception of the 350 °C sample. The higher carbon content observed at 350 °C compared with 500 °C was accompanied by a lower nitrogen content. This may indicate that the proteinaceous macromolecular structure of wool, which contains substantial amounts of nitrogen, was not fully decomposed at 350 °C. At higher temperatures, increased thermal decomposition of the wool structure promoted the transfer of nitrogen- and sulphur-containing components into the volatile products, resulting in higher concentrations of these elements in the corresponding bio-oils.

#### 2.2.5. GC-MS of Bio-Oils

A comparison was first made between the two layers of the oil sample obtained at 350 °C (Figure S4). Several compounds were common to both layers, including the peaks at 20.2 min (p-cresol), 21.2 min (4-piperidinone, 2,2,6,6-tetramethyl-), and 26.8 min (indole). These compounds were also detected in samples produced at higher temperatures and in the fixed-bed experiments. Their higher abundance in the top oil layer suggests that they were not responsible for the distinct appearance of the bottom layer. The peak at 15.3 min was assigned to 5-(2-bromoethyl)-bicyclo[2.2.1]hept-2-ene (C_8_H_11_Br), which accounted for more than 70% of the GC–MS peak area of the bottom layer. Bromine-containing compounds were detected in most of the samples, although generally at low concentrations, typically accounting for less than 2% of the total GC–MS peak area.

The existence could be related to the dyes used in the processed wool, although this could not be confirmed. The dominance of this bromine-containing compound, together with its polarity, may explain the separation of the oil into two distinct layers: a non-polar oily phase at the top and a more polar phase at the bottom. The compound is non-aromatic and contains carbon–carbon double bonds. As discussed previously, the wool was not completely decomposed at 350 °C, and the breakdown of intermolecular bonds, such as hydrogen bonds, was therefore expected to be dominant. At higher temperatures, these smaller linear compounds may undergo further decomposition and transformation into more complex compounds, acting as intermediate products in the overall pyrolysis process. Although pyrolysis at 350 °C was insufficient for complete decomposition of the wool waste, a staged pyrolysis process incorporating an initial low-temperature stage could potentially provide an opportunity to recover bromine-containing compounds. Further investigation would be required to confirm this mechanism and assess the feasibility of such an approach. To investigate the effect of increasing the temperature on the oil produced in the auger reactor and to compare its behaviour with that of the fixed-bed reactor, the ten most abundant compounds identified at 800 and 900 °C in both systems were compared (Table S1). A notable difference was the shift in the dominant chemical functionality in the auger reactor oils from phenolic compounds towards ketones. In contrast, p-cresol was the most abundant compound in the fixed-bed oil. Its formation may be associated with the further transformation of benzocyclobuten-1(2H)-one through carbonyl reduction followed by hydroxyl-group addition. Comparison of the auger reactor products at 800 and 900 °C also indicated a reduction in product complexity with increasing temperature, accompanied by an increase in the relative abundance of the most dominant compounds. At 900 °C, the ten most abundant compounds accounted for more than 65% of the bio-oil, compared with less than 48% at 800 °C. This trend suggests that increasing the temperature promoted the further degradation or conversion of a wider range of compounds into a smaller number of more dominant products.

### 2.3. Pyrolysis Mechanism

**Figure 3:**
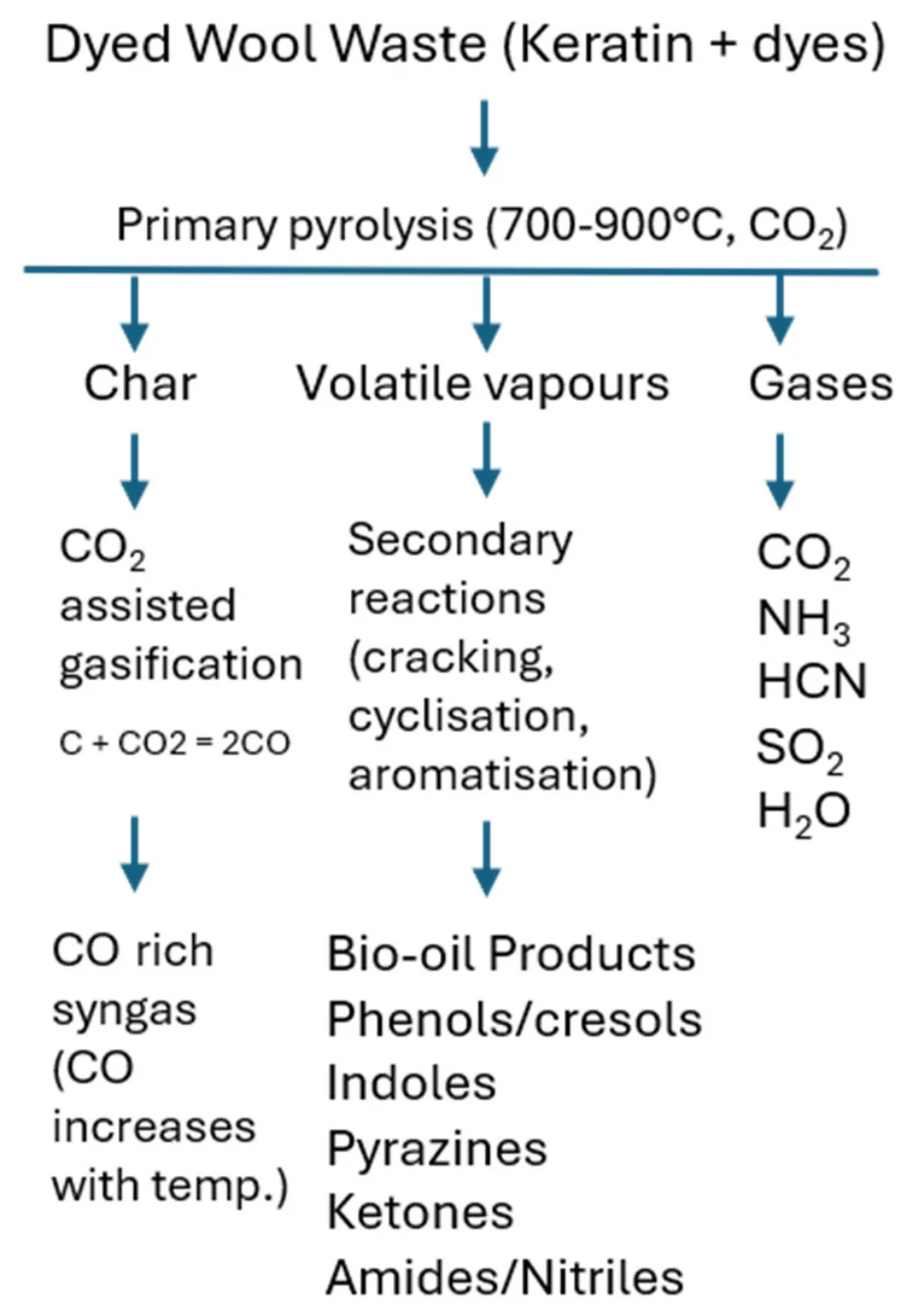
Proposed mechanism for pyrolysis of dyed wool waste under a CO_2_ atmosphere.

The pyrolysis of dyed wool waste (Figure 3) under a CO_2_ atmosphere proceeds through the thermal decomposition of keratin and dye molecules, producing char, gases, and bio-oil. Initially, the peptide, disulfide, and carboxyl groups break down, releasing CO_2_, NH_3_, HCN, SO_2_, and various oxygenated and nitrogenated intermediates. As the temperature increases (900 °C), the CO_2_ generated from decarboxylation reacts with the carbonaceous char through the reverse Boudouard reaction (C + CO_2_ → 2CO), leading to a significant increase in the CO concentration (63%) and the formation of a CO-rich syngas. Thus, CO_2_ acts not merely as an inert carrier gas but as a reactive gasification agent. Simultaneously, secondary cracking, reforming, cyclisation, and aromatisation reactions convert the volatile intermediates into phenols, cresols, indoles, pyrazines, ketones, and other nitrogen-containing compounds. The aromatic dyes contribute substantially to the formation of phenolic products, while amino acid-derived species promote the formation of nitrogen heterocycles. The increasing abundance of cresols with temperature suggests the progressive decomposition and de-sulfonation of dye molecules. The simultaneous increase in SO_2_ supports this mechanism. Higher temperatures also favour aromatic condensation and heterocycle formation, explaining the increase in indoles at 900 °C. Compared with N_2_, CO_2_ actively participates in secondary reactions, enhancing phenol production, reducing methoxy compounds, and increasing product diversity, demonstrating that CO_2_ functions not only as a carrier gas but also as a reactive gasification and reforming agent during pyrolysis.

## 3. Materials and Methods

### 3.1. Materials

The processed waste wool was supplied by Harris Tweed Authority (Stornoway, UK) and was received in two forms: as pieces with a size ranging from 1 by 1 to 10 by 20 cm and wool strings (loose waste). Before the tests, the wool waste was shredded into pieces smaller than 1 × 2 cm. The moisture and ash contents of the waste were 8 wt% and 0.5 wt%, respectively.

### 3.2. Pyrolysis Experiments

Parts of the experimental work presented in this study were originally undertaken as part of the doctoral thesis of Kalateh (2021) [31]. Although these results were documented in the thesis, they have not been previously published in a peer-reviewed scientific journal. The present manuscript provides their first archival publication together with a comprehensive analysis of product distribution and elemental partitioning during the pyrolysis of wool textile waste.

#### 3.2.1. Fixed-Bed Experiments

**Figure 4:**
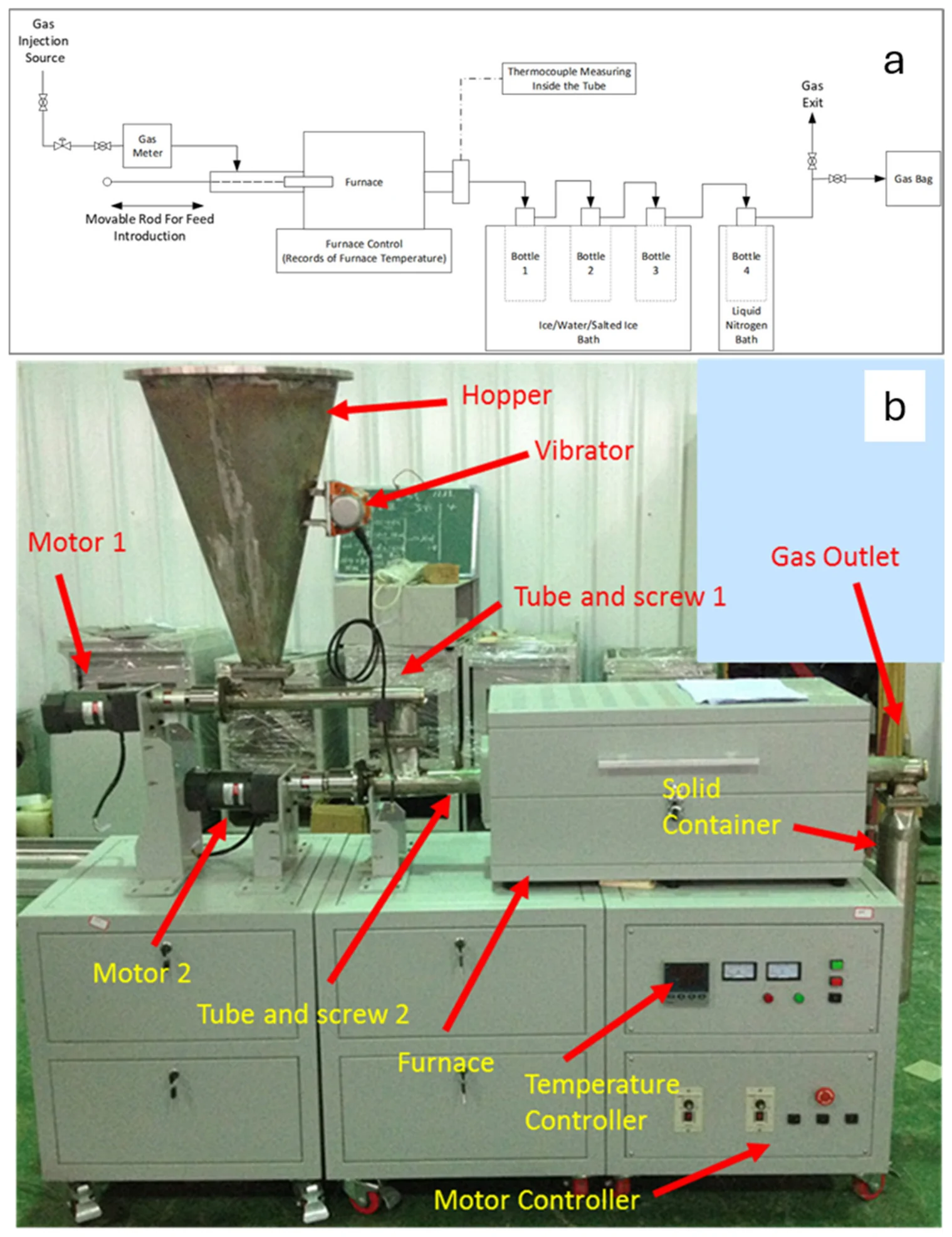
(**a**) Schematic of fixed-bed reactor set-up; (**b**) auger reactor set-up. Runs were run in duplicate with error being <10%.

The pyrolysis set-up used is presented in Figure 4a. It consisted of a semi-fixed bed reactor, a gas distribution section, a tube furnace, and a condensation train. The fixed-bed reactor was composed of a stainless steel SS316 tubing (Swagelok, Aberdeen, UK) with an internal diameter of 2.54 cm and length of 39 cm. The reactor was placed into a tube furnace EVA 1200 (Carbolite Gero Ltd., Hope, UK) with a heated length of 13 cm and a maximum operating temperature of 1000 °C. The gas was distributed and controlled via manometers and valves. The exit was attached to a gas bag to collect the gas for further analysis. The arrangement was equipped with a movable probe to introduce the feed only when the desired temperature within the reactor was reached to obtain isothermal conditions. Additionally, a thermocouple was installed within the reactor to measure the temperature in the proximity of the sample, where variations were on the order of ±8 °C. The empty sample crucible and the condensers were all weighed before the experiments. This weighing procedure was repeated after the experiments to obtain the yield of products and the product distribution. The percentage of gas was obtained by the difference. Consequently, any experimental uncertainties associated with incomplete liquid recovery, solvent evaporation during handling, deposits remaining in the reactor or transfer lines, and weighing errors are incorporated into the calculated gas yield. One gram of feed was loaded on the crucible, and the system was purged with nitrogen or CO_2_ gas for 20 min at 50 mL/min; the reactor was heated up electrically to the desired temperature; the feed was introduced via the movable sample holder into the heated section of the reactor when the desired temperature was reached and held at the desired temperature for 15 min; the condensable gas was collected in four 500 mL Dreschel bottle condensers (Fisher Scientific, Loughborough, UK), three of which were placed in ice (0 °C) and one in liquid nitrogen (−196 °C); the incondensable gases were collected in a 1 L Tedlar gas bag; the solid char product was collected in small sealable plastic bags after weighing the boat; the liquid oil was collected with 10 mL of acetone, which was then evaporated in a fume cupboard. Runs were run in triplicate, with the error being <6%. The experiments were conducted as a comparative feasibility study rather than a statistically designed optimisation study; consequently, no factorial experimental design or inferential statistical analysis was performed.

#### 3.2.2. Auger Reactor Experiments

The set-up used in the experiments is described in Figure 4b. The auger reactor set-up was designed in-house and constructed of stainless steel 310 (SS310) by Zhengzhou (China). The furnace has a 60 mm internal diameter with a heated zone of 60 cm, maximum continuous operating temperature of 1000 °C and a heating rate of up to 20 °C/min. The reactor temperature was monitored by thermocouples inserted into the reactor and was automatically controlled by a PID controller, which regulated the electrical power supplied to the heating elements through an SCR power controller, maintaining the setpoint within ±5 °C. The reactor includes a customised patented feeding system made of a hopper able to hold 5 kg of material with paddles to ensure consistent and smooth feeding, and a dedicated screw feeder (1) to control the amount of waste wool fed to the actual auger reactor (screw feeder 2) [32,33]. Both are equipped with a motor to control the speed of the augers. Anoxic atmosphere was guaranteed by injecting N_2_ into the auger reactor (2) and the hopper. A char collector is located at the end of the auger reactor outside the heater zone. The section of the reactor between the heated zone and the condensation system was heated to 300 °C to minimise the loss of condensable oil species (Figure S5). To condense the condensable fraction of the gas produced, two Quick fit jacketed coil condensers in series were selected, and a 250 mL Schlenk tube was installed at the bottom of each of them to collect the oil. This system gives an overall contact area of 800-centimetre cubes between the oil and the cold water, which was used as the cooling agent. In this arrangement, water was continuously run through the coils while the oil passes around it. The gases were then scrubbed in water before being released to the atmosphere. The condensation system in operation can be seen in Figure S6.

### 3.3. Product Analyses

Elemental analyses were carried out using an Exeter CE-440 Elemental analyser (Exeter Analytical, Coventry, UK) to define the percentages of carbon, hydrogen, and nitrogen in the oil and char products. The GC-MS tests of the oils were carried out using a GC 8000 series equipped with a VG Trio 1000 (VG Systems Limited, Altrincham, UK). The column (length: 30 m, inner diameter: 0.250; film: 0.25 µm) had temperature limits between 40 °C and 300 °C. The oven was programmed to hold at 40 °C for 10 min, ramp at 5 °C/min to 200 °C and hold for 15 min, ramp at 10 °C/min to 240 °C and hold for 15 min, and ramp at 10 °C/min to 260 °C and hold for 10 min. He was used as the carrier gas with a constant flow rate of 1.7 mL/min and an injector split ratio of 1:20 ratio. The end of the column was directly introduced into the ion source detector (VG Trio 1000 series) (VG Systems Limited, Altrincham, UK). Typical mass spectrometer operating conditions were as follows: transfer line 270 °C, ion source 250 °C, and electron energy of 70 eV. The chromatographic peaks were identified according to the NIST library to identify bio-oil components. A PerkinElmer Frontier (PerkinElmer, Shelton, USA) was employed for the FT-IR analyses. As the feedstock originated from industrial textile processing, it may have contained residual processing chemicals, including dyes, finishing agents, and other additives. However, the specific chemical compositions of these residual compounds were not determined in this study. Consequently, the pyrolysis products identified by GC–MS cannot be unequivocally attributed to individual dyes or textile additives. Gas analyses were performed using a MKS Cirrus II Mass Spectrometer (MS) (MKS Instruments UK Ltd, Crewe, UK). The instrument was equipped with a quadrupole analyser incorporating a closed ion source, a triple mass filter and a dual (Faraday and Secondary Electron Multiplier) detector system.

## 4. Conclusions

This study evaluated the pyrolysis of textile wool waste to produce nitrogen-rich biochar and bio-oil under different conditions and using a fixed-bed or an auger reactor. The results indicate that char marketable as biochar with a BET of 190 m^2^/g and C content >70 wt% could be produced at temperatures higher than 700 °C by the pyrolysis of processed wool waste in the presence of nitrogen. The presence of carbon dioxide instead reduced the char yield and surface due to dry reforming. Furthermore, the high temperature biochar had a high nitrogen content (12–15 wt%), low H/C and low O/C, which make it nutrient-rich and stable and consequently usable as fertiliser or as a soil amendment. Overall, there was not a significant difference between the char samples obtained from the fixed-bed reactor and the auger reactor. The main compounds found in the bio-oil were pyrroles, phenolics and indoles, with their quantities increasing proportionally with an increase in temperature. CO_2_ as the carrier gas, compared to nitrogen, favoured phenol and indole generation, while methoxy groups were favoured through the use of nitrogen. Despite low-temperature pyrolysis (350 °C) not being efficient in decomposing the whole wool waste, a staged pyrolysis with an initial low-temperature stage was shown to be effective in separately recovering bromine-rich compounds, thereby improving the quality of the bio-oil. Gas analysis indicated that an increase in temperature largely favoured the production of CO over CO_2_, which makes the gas a potential feedstock for F-T. Water scrubbing was able to remove toxic NOx and SOx from the exhaust gases and to potentially recover 3,3′-thiobis Propanenitrile. Elemental analysis of the bio-oils from the auger reactor showed variations in the carbon, nitrogen, and oxygen contents with temperature changes. Differences in the oil composition between the two layers of samples obtained at lower temperatures highlighted specific compound formations and the potential for staged compound recovery during pyrolysis.

## Figures and Tables

**Table 1:** Temperature effect on product distribution.

	Product Yield, wt%		
Experiment	Char	Oil	Gas
Wool waste-N_2_-800 °C	12.6	31.6	55.8
Wool waste-CO_2_-700 °C	18.0	29.6	52.4
Wool waste-CO_2_-800 °C	12.2	26.3	61.5
Wool waste-CO_2_-900 °C	8.1	12.8	79.1

**Table 2:** Effect of temperature on BET surface (m^2^/g) and elemental composition of chars obtained in the presence of CO_2_.

		Element, wt% (daf)					
	BET	C	H	N	O	H/C (molar)	O/C (molar)
Raw wool waste	/	50.15	7.34	15.91	26.61	1.74	0.40
Wool waste-CO_2_-700 °C	59	75.5	3.2	15.0	6.3	0.505	0.063
Wool waste-CO_2_-800 °C	113	76.8	0.7	11.1	11.4	0.109	0.111
Wool waste-CO_2_-900 °C	190	82.5	0.7	9.9	6.9	0.101	0.063

**Table 3:** Effect of temperature variation on gas composition.

	Product Yield, vol%						
	H_2_O	CO	CO_2_	NH_3_	HCN	C_2_H_7_N	SO_2_
Wool waste-CO_2_-700 °C	4.0	35.7	55.8	1.18	0.66	0.78	0.03
Wool waste-CO_2_-800 °C	6.0	52	36.3	1.52	0.78	0.60	0.05
Wool waste-CO_2_-900 °C	8.1	65.8	20.1	2.0	0.84	0.4	0.3

**Table 4:** Top 10 products in the bio-oil samples to observe the effect of pyrolysis temperature variation.

700 °C		800 °C		900 °C	
**Compound**	Area %	Compound	Area %	Compound	Area %
Phenol, 2-methyl-	9.1	Phenol, 3-methyl-	18.2	*p*-Cresol	18.4
2,4-Imidazolidinedione, 5,5-dimethyl-	7.2	Indole	4.7	2,4-Imidazolidinedione, 5,5-dimethyl-	10.4
4-Piperidinone, 2,2,6,6-tetramethyl-	4.8	Phenol	4.2	Indole	7.3
Pyrrolo[1,2-a] pyrazine-1,4-dione, hexahydro-3-(2-methylpropyl)-	3.4	4-Piperidinone, 2,2,6,6-tetramethyl-	3.9	4-Piperidinone, 2,2,6,6-tetramethyl-	5.5
β-D-Glucopyranose, 1,6-anhydro-	3.4	2-Propen-1-amine, *N*,*N*-bis(1-methylethyl)-	3.0	Phenol	3.7
Phenol	3.3	4-Aminoresorcinol	2.4	4-Aminoresorcinol	2.4
5,10-Diethoxy-2,3,7,8-tetrahydro-1H,6H-dipyrrolo[1,2-a:1′,2′-d] pyrazine	3.2	5,10-Diethoxy-2,3,7,8-tetrahydro-1*H*,6*H*-dipyrrolo[1,2-a:1′,2′-d] pyrazine	2.4	4-Piperidinone, 4-Piperidinone, 2,2,6,6-tetramethyl-	2.1
2,4-Imidazolidinedione, 5-(2-methylpropyl)-, (*S*)-	2.8	Pyrrolo[1,2-a] pyrazine-1,4-dione, hexahydro-	2.3	Phenol, 4-ethyl-	1.6
Indole	2.8	Cyclopropylamine, *N*-isobutylidene-	2.1	Aziridine, 1,2-diisopropyl-3-methyl-, *trans*-	1.5
Pyrrolo[1,2-a] pyrazine-1,4-dione, hexahydro-	2.2	Hexahydropyrrolizin-3-one	2.0	2-Pentanone, 4-hydroxy-4-methyl-	1.5
*p*-Cresol	1.9	Aziridine, 1,2-diisopropyl-3-methyl-,	1.9	Benzofuran, 2,3-dihydro-	1.3
Overall	44.0	Overall	47.0	Overall	55.7

**Table 5:** Elemental analysis of bio-oil samples obtained using the auger reactor.

	Element, wt%			
	C	H	N	O
Wool waste-CO_2_-350 °C (top)	60.1	7.9	11.7	20.3
Wool waste-CO_2_-350 °C (bottom)	59.9	7.8	11.1	21.2
Wool waste-CO_2_-500 °C	54.5	7.7	14.2	23.6
Wool waste-CO_2_-800 °C	68.8	5.2	14.1	11.9

## Data Availability

The authors will make the materials available upon request.

## Supplementary Materials

The following supporting information can be downloaded at: https://www.mdpi.com/article/10.3390/molecules31162816/s1, Figure S1: (a) Wool before pyrolysis, after pyrolysis in the ceramic boat and char at 3 different temperatures (right); (b) char appearance after continuous test in auger reactor at 350 °C; (c) biooil obtained at 350 °C (left) and 800 °C (right). Figure S2: The effect of back flow and location of the feed on the screw on the product differentiation. Figure S3. GC-MS curve for the scrubbing water obtained at 800 °C. Figure S4. Comparison of GC-MS results obtained for top and bottom layers of pyrolysis oil sample at 350 °C. Figure S5. Sleeve, the inner second thermal isolation layer on top of mineral wool (right), third layer of isolation, aluminium tape (left). Figure S6: Appearance of gas and water as it enters the condensation train and gas scrubbing system during the experiment. Table S1: Effect of increasing temperature on oil composition from auger reactor (AR) and fixed bed (FB).
