# Supplementary material for: Upcycling Wool Textile Waste by Slow Pyrolysis to Recover Nitrogen-Rich Bio-Oil and Bio-Char and CO-Rich Gas Using Bespoke Auger Reactor
Source: Molecules. 2026 Aug 13;31(16):2816. doi: 10.3390/molecules31162816 (PMC13515442; doi:10.3390/molecules31162816)
Supplement: Supplementary file 1 [file molecules-31-02816-s001.zip › molecules-4470533-supplementary.pdf]

Supplementary material

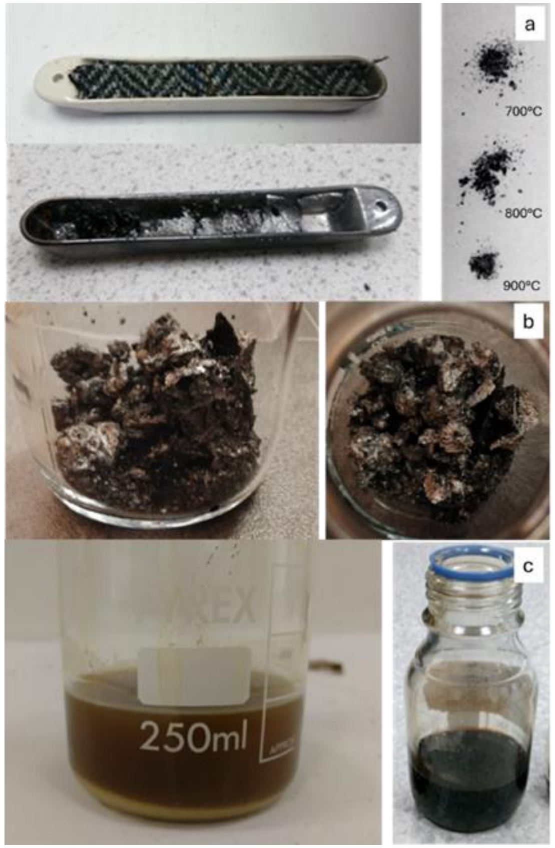

Figure S1: (a) Wool before pyrolysis, after pyrolysis in the ceramic boat and char at 3 different temperatures (right); (b) char appearance after continuous test in auger reactor at 350 °C; (c) biooil obtained at 350 °C (left) and 800 °C (right).

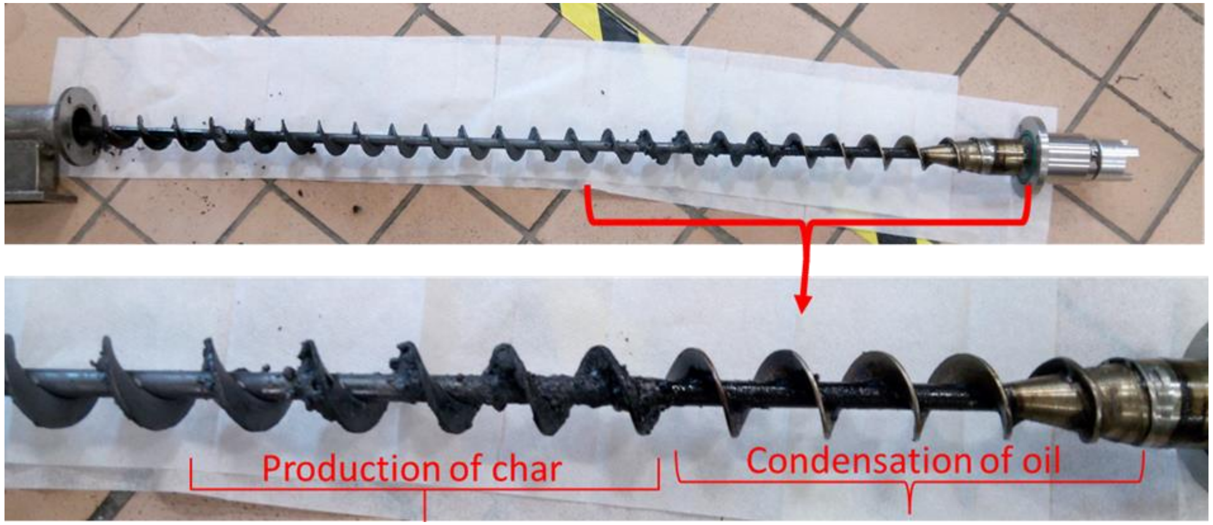

Figure S2: The effect of back flow and location of the feed on the screw on the product differentiation.

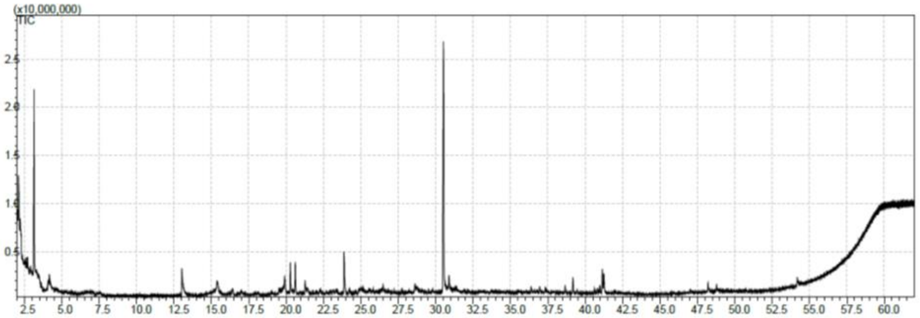

Figure S3. GC-MS curve for the scrubbing water obtained at 800°C.

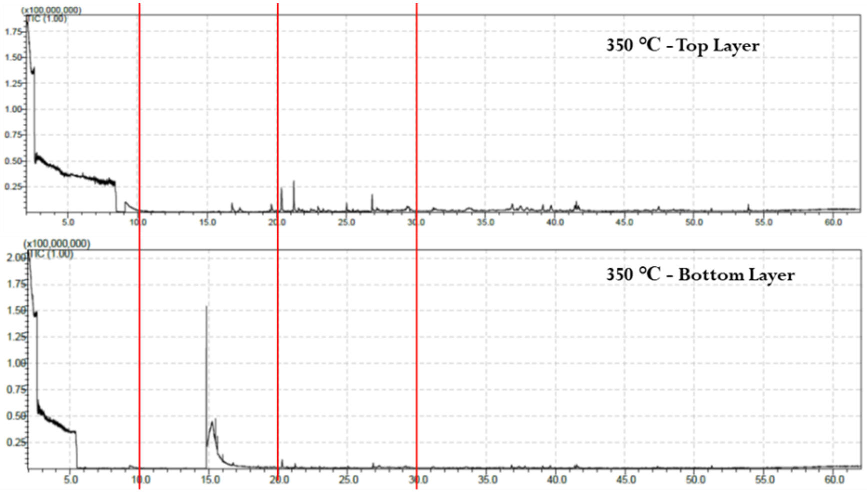

Figure S4. Comparison of GC-MS results obtained for top and bottom layers of pyrolysis oil sample at 350°C.

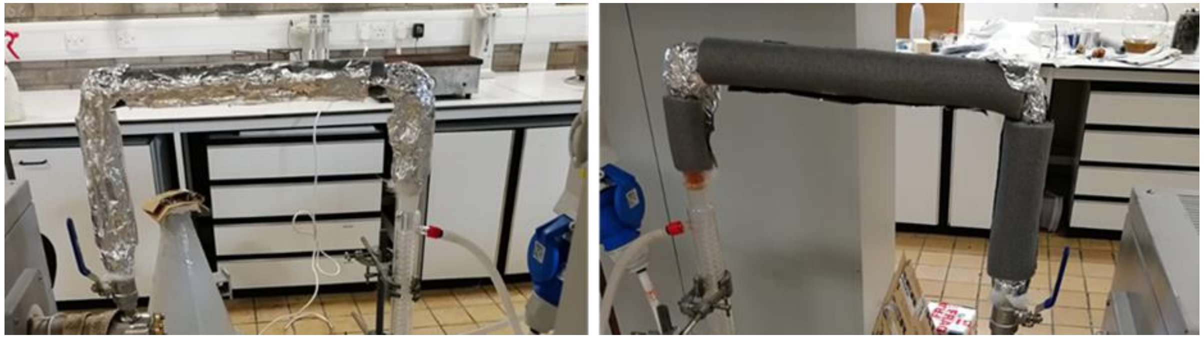

Figure S5. Sleeve, the inner second thermal isolation layer on top of mineral wool (right), third layer of isolation, aluminium tape (left).

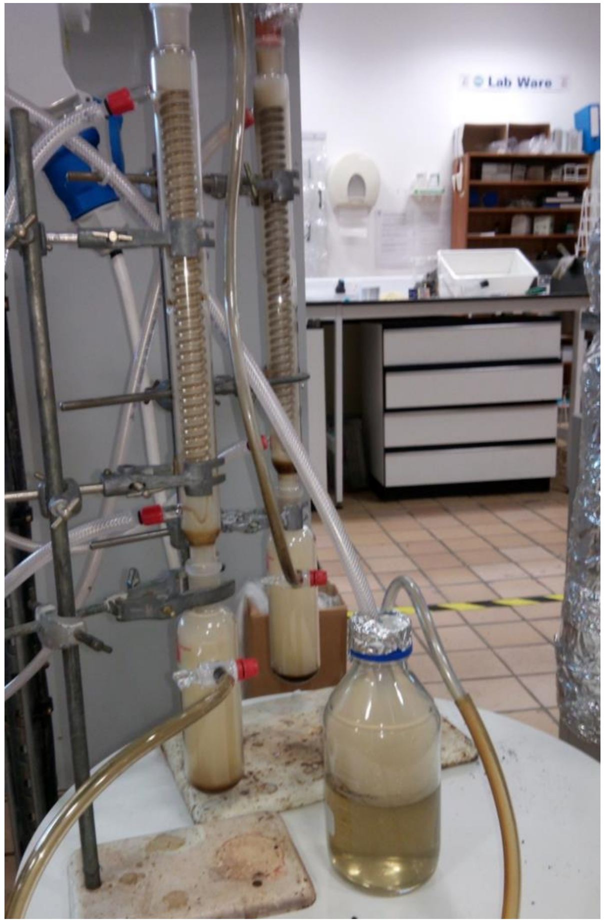

Figure S6: Appearance of gas and water as it enters the condensation train and gas scrubbing system during the experiment.

Table S1: Effect of increasing temperature on oil composition from auger reactor (AR)and fixed bed (FB).

800℃ (FB)		800℃ (AR)		900℃ (FB)		900℃ (AR)	
Compound	Area %	Compound	Area %	Compound	Area %	Compound	Area %
Phenol,3-methyl-	18.2	Benzocyclobuten-1(2H)-one	10.9	p-Cresol	18.4	4-Isobutyl-2-pyrrolidinone	32.48
Indole	4.7	1-Naphthalenecarbonitrile	6.9	2,4-Imidazolidinedione, 5,5-dimethyl-	10.4	Cycloprop[a]indene, 1,1a,6,6a-tetrahydro-	6.45
Phenol	4.2	4-Piperidinone, 2,2,6,6-tetramethyl-	6.1	Indole	7.3	Bis-(2,2,6,6-tetramethyl-piperidin-4-yl)-amine	4.58
4-Piperidinone, 2,2,6,6-tetramethyl-	3.9	Phenylpropynal	5.5	4-Piperidinone, 2,2,6,6-tetramethyl-	5.5	1-Naphthalenecarbonitrile	3.68
2-Propen-1-amine, N, N-bis(1-methylethyl)-	3.0	1H-Pyrrole-2-carbonitrile	3.2	Phenol	3.7	1,2,4,5-Tetrazin-3-amine, 6-methyl-	3.40
4-Aminoresorcinol	2.4	Azulene	3.0	4-Aminoresorcinol	2.4	Isoquinoline	2.64
5,10-Diethoxy-2,3,7,8-tetrahydro-1H,6H-dipyrrolo[1,2-a:1',2'-d] pyrazine	2.4	Isoquinoline	2.8	4-Piperidinone, 4-Piperidinone, 2,2,6,6-tetramethyl-	2.1	Hexahydro-1,3,5-trinitroso-1,3,5-triazine	2.63
Pyrrolo[1,2-a] pyrazine-1,4-dione,hexahydro-	2.3	Biphenylene	2.4	Phenol, 4-ethyl-	1.6	p-Cresol	2.28
Cyclopropylamine, N-isobutylidene-	2.1	Phenanthridine	2.3	Aziridine, 1,2-diisopropyl-3-methyl-, trans-	1.5	2-Ethynyl pyridine	2.21
Hexahydropyrrolizin-3-one	2.0	Anthracene	2.2	2-Pentanone, 4-hydroxy-4-methyl-	1.5	Pyrene	2.15
Aziridine, 1,2-diisopropyl-3-methyl-,	1.9	Benzo[h]quinoline	2.0	Benzofuran, 2,3-dihydro-	1.3	Phenanthrene	2.04
